# The selective antifungal activity of *Drosophila melanogaster* metchnikowin reflects the species-dependent inhibition of succinate–coenzyme Q reductase

**DOI:** 10.1038/s41598-017-08407-x

**Published:** 2017-08-15

**Authors:** Mohammad-Reza Bolouri Moghaddam, Thomas Gross, Annette Becker, Andreas Vilcinskas, Mohammad Rahnamaeian

**Affiliations:** 1 Institute for Insect Biotechnology, Justus Liebig University of Giessen, Heinrich-Buff-Ring 26-32, D-35392 Giessen, Germany; 2 Fraunhofer Institute for Molecular Biology and Applied Ecology, Department of Bioresources, Winchester Strasse 2, D-35394 Giessen, Germany; 3 Institute of Botany, Justus Liebig University of Giessen, Heinrich-Buff-Ring 38, D-35392 Giessen, Germany

## Abstract

Insect-derived antifungal peptides have a significant economic potential, particularly for the engineering of pathogen-resistant crops. However, the nonspecific antifungal activity of such peptides could result in detrimental effects against beneficial fungi, whose interactions with plants promote growth or increase resistance against biotic and abiotic stress. The antifungal peptide metchnikowin (Mtk) from *Drosophila melanogaster* acts selectively against pathogenic Ascomycota, including *Fusarium graminearum*, without affecting Basidiomycota such as the beneficial symbiont *Piriformospora indica*. Here we investigated the mechanism responsible for the selective antifungal activity of Mtk by using the peptide to probe a yeast two-hybrid library of *F. graminearum* cDNAs. We found that Mtk specifically targets the iron-sulfur subunit (SdhB) of succinate–coenzyme Q reductase (SQR). A functional assay based on the succinate dehydrogenase (SDH) activity of mitochondrial complex II clearly demonstrated that Mtk inhibited the SDH activity of *F. graminearum* mitochondrial SQR by up to 52%, but that the equivalent enzyme in *P. indica* was unaffected. A phylogenetic analysis of the SdhB family revealed a significant divergence between the Ascomycota and Basidiomycota. SQR is one of the key targets of antifungal agents and we therefore propose Mtk as an environmentally sustainable and more selective alternative to chemical fungicides.

## Introduction

Insects are well protected against pathogens by an immunity-related arsenal of effector molecules including antimicrobial peptides (AMPs). Some AMPs are active against a broad spectrum of microbes, whereas the activity of others is restricted to certain types of bacteria or fungi. Insects produce a large number of antifungal peptides to protect them against fungal pathogens and parasites, and these peptides often interact with intracellular targets to inhibit key physiological processes such as DNA and protein synthesis, cell cycle progression and metabolic activity^1–3^. AMPs therefore offer significant potential as leads for the development of drugs and biocides, but the mechanisms of action must first be understood^4, 5^. A small number of natural antifungal peptides have been characterized in this regard, including termicin from termites^6^, heliomicin from the tobacco budworm *Heliothis virescens* ^7^, gallerimycin from larvae of the greater wax moth *Galleria mellonella* ^8^, and drosomycin and metchnikowin (Mtk) from *Drosophila melanogaster* ^9, 10^. The 26-residue proline-rich linear peptide Mtk is induced following microbial infection in *D. melanogaster* and its activity is remarkably specific. Whereas the activity of other antifungal peptides can affect both pathogens and beneficial endophytes, Mtk acts specifically against pathogenic Ascomycota such as *Fusarium graminearum* and *Blumeria graminis* f. sp. *hordei*, but is inactive against beneficial endophytic Basidiomycota such as *Piriformospora indica*, when expressed in barley^11^. This is particularly important because plants benefit from their mutual interactions with fungal endophytes^12^. Although the precise basis of this specificity is not understood, previous observations suggest that Mtk inhibits the ability of susceptible fungi to suppress plant defense responses^11^. A recent study revealed that Mtk interferes with *F. graminearum* cell wall biosynthesis by targeting the β(1,3)-glucanosyltransferase Gel1, which is responsible for β(1,3)-glucan chain elongation in the cell wall^13^.

AMPs often have multiple targets to reduce the likelihood of emerging microbial resistance. We therefore sought additional Mtk intracellular targets by probing a yeast two-hybrid library of *F. graminearum* cDNAs with an artificial Mtk peptide, revealing a specific interaction with the iron-sulfur subunit (SdhB) of succinate–coenzyme Q reductase (SQR). The holoenzyme is a heterotetramer comprising two hydrophilic subunits (flavoprotein SdhA and iron-sulfur protein SdhB) and two hydrophobic subunits (SdhC and SdhD). SdhA contains the cofactor flavin adenine dinucleotide (FAD) and a succinate binding site, whereas SdhB contains three iron-sulfur clusters, and SdhC and SdhD are the membrane anchor subunits^14^. SQR is a key enzyme in both the Krebs cycle (also known as the citric acid cycle or tricarboxylic acid cycle) and the electron transport chain^15^, both of which are required for energy generation. In the Krebs cycle, SQR catalyzes the oxidation of succinate to fumarate via the reduction of ubiquinone to ubiquinol^16^. The resulting electrons enter the respiratory chain complex III, reducing oxygen to water and thus providing the electrochemical gradient across the mitochondrial inner membrane which is needed for ATP synthesis.

We therefore investigated whether Mtk can selectively inhibit mitochondrial SQR (complex II) activity in *F. graminearum* but not in *P. indica*, based on our hypothesis that the specific activity of Mtk against Ascomycota reflects the selective inhibition of this enzyme. We also conducted a phylogenetic analysis of SdhB homologs in different Ascomycota and Basidiomycota to determine whether the enzyme has diverged in these two fungal phyla.

## Results

### Bait expression, auto-activation and toxicity tests

**Figure 1:**
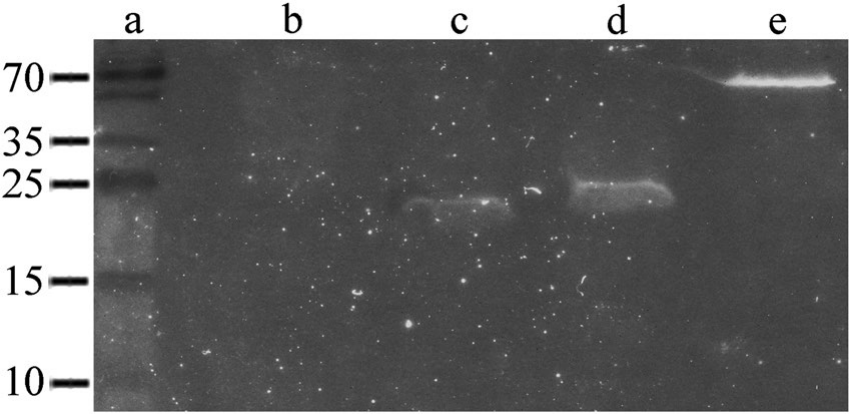
Western blotting to confirm the expression of Mtk in yeast. The expression of Mtk in yeast strain Y2HGold was confirmed by western blotting using a Myc-specific antibody. (**a**) Size marker (kDa). (**b**) Negative control (Y2HGold). (**c**) Empty pGBKT7 vector. (**d**) pGBKT7::Mtk. (**e**) Positive control pGBKT7::P53.

The in-frame cloning of sequences encoding Mtk, the GAL4 DNA-binding domain (DNA-BD) and the Myc epitope tag was verified by sequencing and the *in vivo* expression of the bait protein was confirmed by western blotting (Fig. 1). An auto-activation test was carried out to ensure that the bait does not activate the transcription of the reporter genes in the absence of an interacting partner. The absence of growth on single-dropout plates lacking tryptophan and supplemented with X-α-Gal and aureobasidin A (SDO/–Trp/X/A) confirmed the absence of auto-activation (data not shown). We also compared the growth of colonies transformed with either the pGBKT7 empty vector or pGBKT7::Mtk on SDO/–Trp plates to confirm that Mtk was not independently toxic towards yeast cells (data not shown).

### Construction of the yeast two-hybrid libraries

*F. graminearum* cDNA was synthesized from total hyphal RNA. To ensure the discovery of rare transcripts, we normalized the cDNA to equalize the prevalence of abundant and rare transcripts and then size selected the cDNA fragments by spin-column chromatography to yield a population of fragments in the size range 200 bp to 4 kbp. We then directly constructed the yeast two-hybrid library in the *Saccharomyces cerevisiae* prey strain Y187. Competent Y187 yeast cells were co-transformed with the normalized cDNA and the pGADT7-Rec vector, and the transformants were pooled after incubation for 3–5 days at 30 °C on single-dropout plates lacking leucine (SDO/–Leu).

### Confirmation that Mtk targets the iron-sulfur subunit (SdhB) of SQR

More than 9 × 10^5^ transformants were screened after mating the bait and prey strains in 2× yeast peptone dextrose adenine (YPDA) medium. We evaluated zygote formation after 24 h and initially plated the cells onto low-stringency double-dropout medium supplemented with X-α-Gal and aureobasidin A (DDO/X/A), resulting in the selection of 153 diploids. After 3–5 days, the blue colonies were re-streaked on high-stringency quadruple-dropout medium supplemented with X-α-Gal and aureobasidin A (QDO/X/A) resulting in the selection of 54 genuine interactions. We verified the positive interaction candidates by co-transforming yeast with the corresponding prey plasmid and either pGBKT7::Mtk or the empty pGBKT7 vector. The resulting diploids were plated onto DDO/X and QDO/X/A media. Blue colonies that survived on both media were recovered, and the open reading frames fused to the GAL4 activation domain (AD) were sequenced and used as BLAST queries. Nine candidates were identified, including a sequence which shared 83% identity with the full-length iron–sulfur subunit (SdhB) of SQR in *F. graminearum* (NCBI reference sequence: XM_011325868.1).

### Interaction between Mtk and SdhB in BHK-21 cells

**Figure 2:**
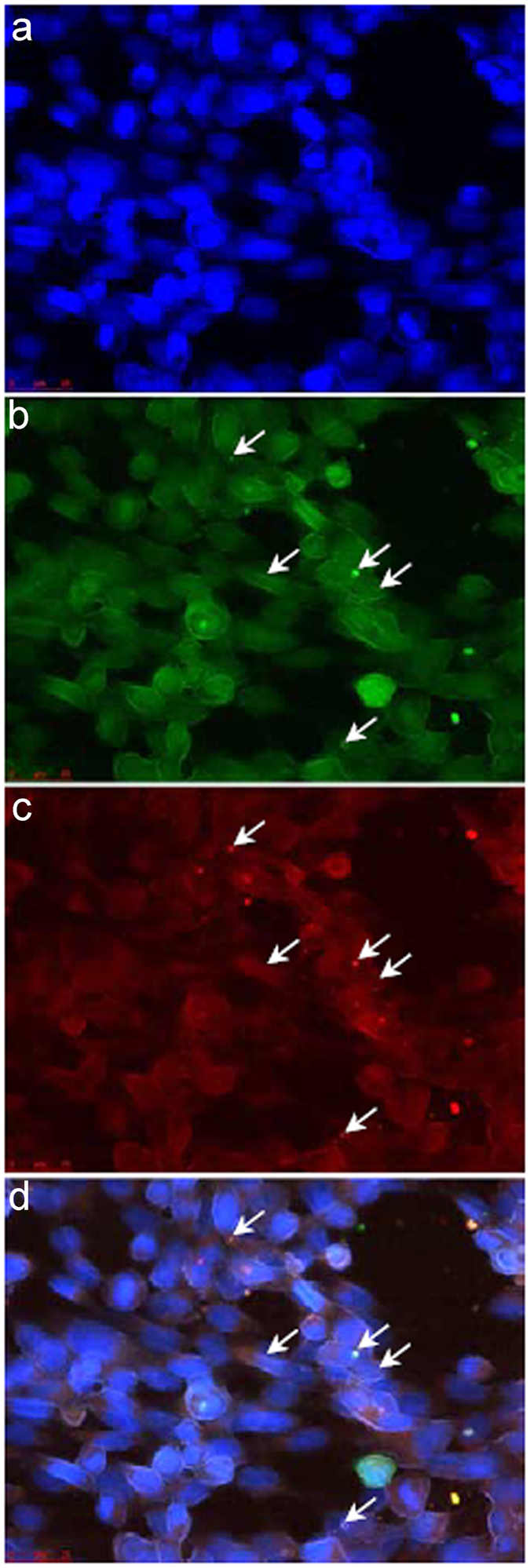
Testing the interaction between Mtk and SdhB in BHK-21 cells. The interaction between Mtk and SdhB was tested using the F2H assay 24 h after the transfection of BHK-21 cells. (**a**) DAPI channel. (**b**) GFP channel. (**c**) RFP channel. (**d**) Merged. The presence of both green and red spots (arrows) in the GFP and RFP channels, respectively, shows the interaction between Mtk and SdhB as confirmed by the presence of both green and red spots. Scale bars = 25 µm.

The interaction between Mtk and SdhB was independently tested in mammalian BHK-21 cells by performing a fluorescent two-hybrid (F2H) assay, in which the co-localization of green fluorescent protein (bait-GFP) and red fluorescent protein (prey-RFP) in the nucleus of co-transfected BHK-21 cells represents a genuine interaction whereas the presence of a green spot alone indicates a lack of interaction. Accordingly, we observed the co-localization of the Mtk-GFP and SdhB-RFP spots in the nucleus (Fig. 2). However, the number of cells supporting the interaction was low, suggesting that the F2H assay was not ideal for the verification of this interaction.

### Interaction between Mtk and SdhB in tobacco cells

To address the limitations of the F2H assay, the interaction between Mtk and SdhB was also tested using bimolecular fluorescence complementation (BiFC)^17, 18^. Each protein was fused to either the N-terminus or C-terminus of yellow fluorescent protein (YFP) thus producing four fusion proteins. All four constructs were tested for auto-activation in the presence of YFP without a fusion partner (YFP_Cempty_ or YFP_Nempty_). Although the SdhB constructs showed no YFP fluorescence in the presence of YFP_Cempty_ or YFP_Nempty_, the Mtk constructs produced YFP fluorescence in both the cytoplasm and nucleus in the presence of the appropriate YFP construct without a fusion partner (Fig. S1). Interestingly, when Mtk and SdhB were co-expressed, the YFP fluorescence was neither robust nor repeatable (Fig. S1), indicating that the BiFC assay is also unsuitable to verify the Mtk/SdhB interaction.

### Mtk reduces SDH activity in *F. graminearum* but not in *P. indica*

**Figure 3:**
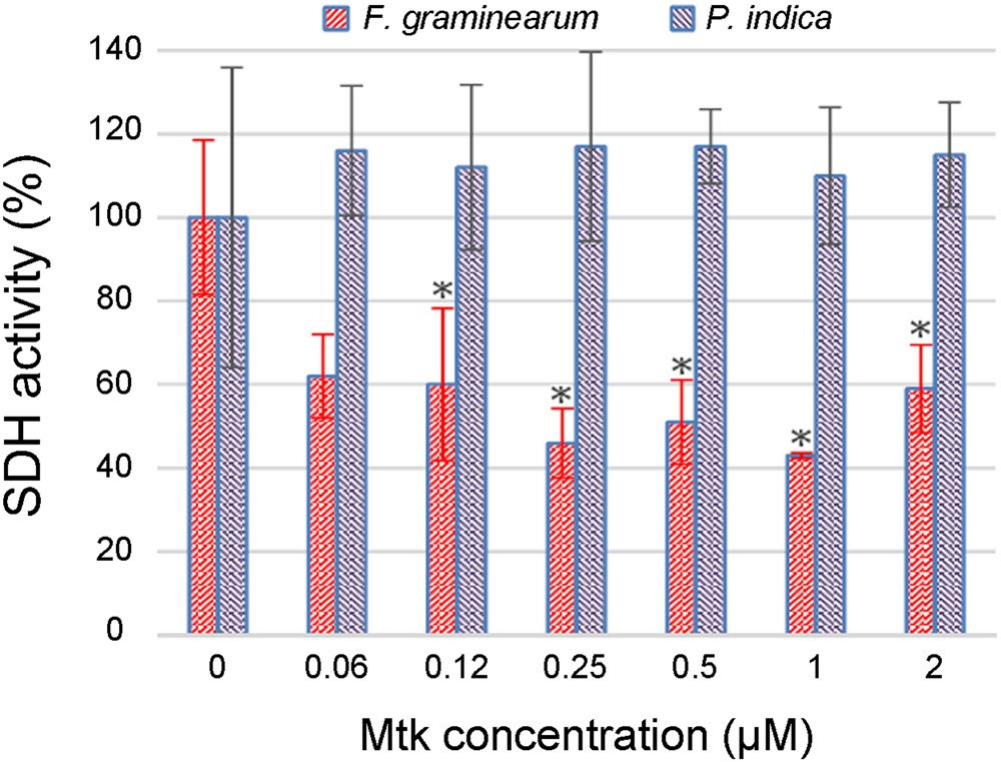
Mtk-dependent loss of SDH activity in *Fusarium graminearum* SQR but not in *Piriformospora indica* SQR. Mitochondria from *F. graminearum* and *P. indica* were incubated in 0.06, 0.12, 0.25, 0.5, 1 and 2 µM of Mtk in triplicate. Significant reductions in *F. graminearum* SDH activity were observed compared to the control (mitochondria without Mtk) but no reduction was observed in *P. indica*. The data are the means of three independent experiments.

The indecisive results obtained using the F2H and BiFC interaction systems made it necessary to confirm the interaction between Mtk and SdhB using a functional assay. We therefore measured succinate dehydrogenase (SDH) activity to investigate the effect of Mtk on the SDH activity of mitochondrial SQR, and this also highlighted the specificity of Mtk against Ascomycota and the absence of activity in Basidiomycota. Protoplasts were prepared from the hyphae of *F. graminearum* and *P. indica*, and the mitochondria were isolated and then used as the source of SQR. The IC_50_ of Mtk against *F. graminearum* is 1 µM^11^ so we exposed the mitochondria from both species to Mtk at concentrations of 2, 1, 0.5, 0.25, 0.12 and 0.06 µM. All concentrations except the lowest caused significant reductions in the mitochondrial SDH activity of *F. graminearum* but not *P. indica* (Fig. 3).

### Phylogenetic analysis of fungal SQR sequences

**Figure 4:**
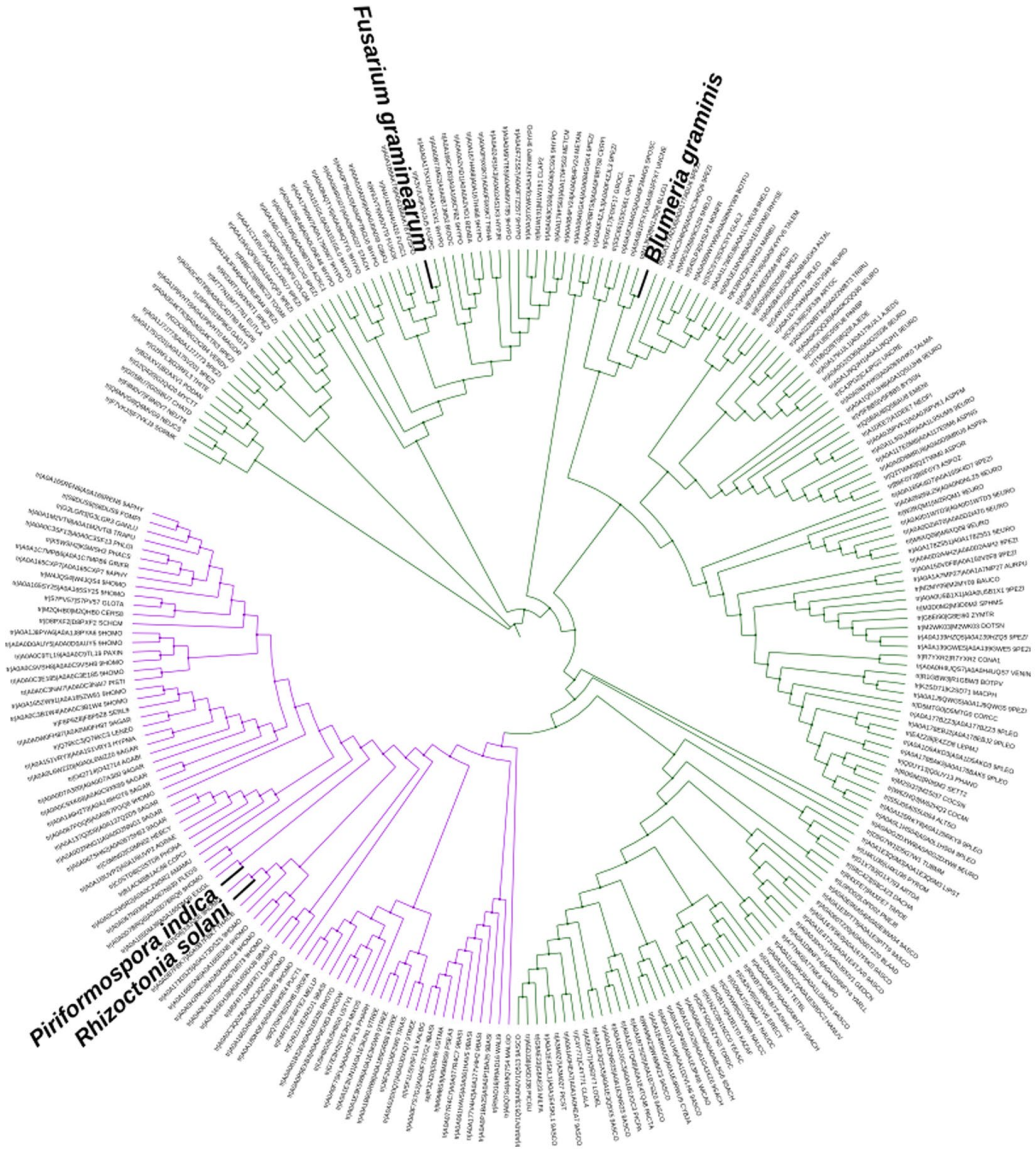
Phylogenetic analysis of SdhB homologs in Ascomycota and Basidiomycota. The phylogenetic tree was built using iTOL on the EMBL-EBI platform (http://itol.embl.de/), showing the evolutionary distance among SdhB homologs in Ascomycota and Basidiomycota. The Basidiomycota clade (represented by *Piriformospora indica* and *Rhizoctonia solani*) is shown in purple, revealing significant divergence from the Ascomycota SdhB sequences (represented by *Fusaium graminearum* and *Blumeria graminis* f. sp. *hordei*). The discrimination between SdhB homologs of Ascomycota and Basidiomycota provides a basis for the selective antifungal activity of Mtk in Ascomycota. For the list of sequences see the Supplementary Information.

The specific activity of Mtk was investigated further by aligning the SdhB amino acid sequences from a range of Ascomycota and Basidiomycota. As shown in Fig. 4, the Basidiomycota SdhB sequences (represented by *P. indica* and *Rhizoctonia solani*) have diverged significantly from the Ascomycota SdhB sequences (represented by *F. graminearum* and *B. graminis* f. sp. *hordei*) and formed a separate clade (shown in purple) providing a potential explanation for the specific activity of Mtk against Ascomycota SQRs.

## Discussion

Insect AMPs have often been shown to act against phytopathogenic fungi, and could therefore be suitable for expression as recombinant peptides in transgenic plants to reduce yield and quality losses. However, the nonspecific antifungal effects of many such peptides limit their plant protection applications because most crops establish beneficial mutualistic interactions with fungal endophytes, which promote growth by facilitating access to water and nutrients, or by inhibiting other pathogens^19^. The deployment of AMPs with selective activity against pathogenic fungi offers an environmentally sustainable alternative to hazardous chemical fungicides, particularly given the emergence and spread of fungicide resistance. The proline-rich antifungal peptide Mtk from *D. melanogaster* ^10^ acts selectively against phytopathogenic Ascomycota such as *F. graminearum* without affecting beneficial endophytic Basidiomycota such as *P. indica* ^11^. Our recent findings indicate that this specific activity partly reflects the interaction between Mtk and the Ascomycota β(1,3)glucanosyltransferase Gel1, which is only distantly related to Gel homologs in the Basidiomycota^13^.

The specificity of Mtk against Ascomycota was considered in more detail by screening a yeast two-hybrid library of *F. graminearum* cDNAs using Mtk as the probe, which identified the iron-sulfur subunit of SQR (UniProtKB-I1RNM7) as an additional Mtk target. We used the F2H and BiFC systems to verify the interaction. The advantage of protein–protein interaction assays based on two-hybrid systems rather than *in vitro* biophysical or biochemical methods is their ability to detect weak or transient interactions. Some of these interactions need species-dependent post-translational modifications and/or particular cofactors, therefore verification in mammalian and/or plant cells is highly recommended^20^. The F2H assay reported a positive interaction but only in a few cells, suggesting the ability of BHK-21 cells to support the interaction was limited. Potential explanations include the nuclear targeting of the bait and prey, forcing them to interact in a non-native compartment, or changes in conformation caused by the fluorescent protein tags, obscuring the interaction site^21^. The BiFC assay was also unsatisfactory, which may reflect the non-optimal stoichiometry of the interacting components caused by differences in transformation efficiency by the separate *Agrobacterium tumefaciens* strains, or non-specific reassembly due to the prevention of translational read-through in the native Gateway cloning cassettes^22^. Furthermore, background signals in BiFC assays can obscure weak interactions. We cannot exclude the possibility that the rare Mtk–SdhB interaction events in mammalian and tobacco cells indicate the lack of physical interaction. However, a functional assay demonstrated that Mtk reduces the SDH activity of *F. graminearum* SQR but not that of its *P. indica* homolog (Fig. 3). Therefore, the absence of an expected physical interaction in the F2H and BiFC assays may reflect the requirement for additional components that are present in the *S. cerevisiae* strain used for the original Y2H assay but not in the BHK-21 or tobacco cells, bearing in mind that *S. cerevisiae* is also a member of the Ascomycota.

The mitochondrial enzyme SQR, which contains three further subunits in addition to the iron-sulfur subunit SdhB, plays an important role in both the Krebs cycle and the mitochondrial electron transport chain, both of which are essential for oxidative phosphorylation. SQR catalyzes the oxidation of succinate to fumarate in the mitochondrial matrix, which results in the reduction of ubiquinone to ubiquinol in the mitochondrial inner membrane. SdhB, which is located between SdhA and the two transmembrane subunits SdhC and SdhD, contains three iron-sulfur clusters that are required for tunneling the electrons through the complex. Coenzyme Q accepts the electrons from complexes I and II, and carries them to complex III, from where they are diverted to reduce the ubiquinone pool. The reducing equivalents reduce superoxide anions that accumulate from either exogenous sources or the respiratory chain^23^. In contrast to other dehydrogenases in the Krebs cycle, SQR does not transport the succinate-derived electrons to soluble NAD^+^ intermediates but to the ubiquinone pool of the respiratory chain as an electron sink to provide antioxidants in the mitochondrial inner membrane^24^. We found that Mtk diminishes the overall mitochondrial SDH activity by up to 52% (Fig. 3), which may lead to (i) higher levels of reactive oxygen species such as superoxide due to the depleted ubiquinone pool, and (ii) loss of the electron gradient, thus compromising the survival of the fungus. The inhibition of SdhB by Mtk may arrest the delivery of electrons required for the full reduction of ubiquinone to ubiquinol, and may therefore increase oxygen toxicity in the mitochondria. On the other hand, a deficient electrochemical gradient across the mitochondrial inner membrane would inhibit the generation of ATP, which is coupled to the oxidation of nicotinamide adenine dinucleotide (NADH)/FADH_2_ and the reduction of oxygen to water within the respiratory chain^25^. Therefore, the inhibition of SDH activity by Mtk would impose a high fitness cost on *F. graminearum*, explaining the resistance of transgenic barley plants expressing Mtk against this pathogen^11^. The ability of Mtk to inhibit SdhB and thus perturb the Krebs cycle could also disturb the equilibrium between the malate–aspartate shuttle and reducing equivalents, further restricting electron delivery from the mitochondrial inner membrane to the electron transport chain. Mutations in the subunits of mitochondrial complex II therefore increase oxidative stress and decrease longevity^26, 27^.

Interestingly, Mtk did not inhibit the SDH activity of mitochondrial SQR in *P. indica* (Fig. 3), providing a further explanation for the selective activity of Mtk against Ascomycota^11^. Although we cannot comment on the relative activities of SQR enzymes in *F. graminearum* and *P. indica*, the mitochondrial respiratory activity in the latter is so high that it can be detected in colonized plant roots using the SDH assay^12^. Our previous studies have already revealed that the selective activity of Mtk is partly due to the specific interaction between Mtk and the Ascomycota β(1,3)-glucanosyltransferase Gel1, which is only distantly related to the equivalent enzyme in the Basidiomycota^13^. The phylogenetic analysis described herein also revealed a significant divergence between the Ascomycota and Basidiomycota SdhB homologs, which provides a mechanistic basis for the selective inhibition we observed (Fig. 4). Mtk is therefore a highly promising antifungal candidate that is active against pathogenic Ascomycota but not against beneficial endophytic Basidiomycota such as *P. indica* due to its phylum-specific activity against at least two distinct enzymes.

SQRs are useful targets for fungicide development. SDH inhibitors (SDHIs) that occupy either the succinate-binding pocket (e.g. malonate) or the ubiquinone-binding pocket (e.g. carboxamides) are highly effective against diverse fungal species^24, 28^. Amide fungicides target SQR in the mitochondrial respiratory chain^29^ resulting in growth arrest and even cell death by disrupting the mitochondrial Krebs cycle^30^ and interfering with respiration^31^. All SDHIs currently used for crop protection target the ubiquinone-binding pocket, which is structurally defined by the interface among the subunits SdhB, SdhC and SdhD. However, mutations in SQR subunits have conferred SDHI resistance in 14 fungal species thus far^24^, highlighting the need for new antifungal agents with diverse mechanisms of action. Although *F. graminearum* remains susceptible to chemical SDHIs for the time being, mutations that reduce susceptibility have been identified in a number of pathogens including *B. cinerea* ^32, 33^, *Podosphaera xanthii* ^34, 35^, *Alternaria* spp.^36, 37^ and *Sclerotinia sclerotiorum* ^38^. Mtk could help to prevent or at least delay the emergence of resistant fungal pathogens because resistance would require the simultaneous mutation of multiple targets.

In conclusion, we have shown that Mtk attacks *F. graminearum* using at least two distinct mechanisms. First the integrity of the cell wall is compromised when Mtk interacts with the β(1,3)glucanosyltransferase Gel1 to inhibit the synthesis of cell wall polymers, and then the general metabolic fitness of the fungus is targeted by inhibiting the SDH activity of mitochondrial SQR, resulting in suboptimal energy generation. The fungus suffers a loss of fitness due to the combined impact of a weak cell wall, compromised energy generation, oxidative stress and slow metabolism. Mtk represents an ideal lead for the development of environmentally sustainable fungicides due to its selectivity and multiple intracellular targets, which reduces the impact on beneficial fungi and discourages the emergence of resistant pathogens.

## Materials and Methods

### Expression constructs

The coding sequence of *D. melanogaster* Mtk (isoform HRH) was amplified using the primers shown in Table 1. The PCR amplicon was digested with EcoRI and BamHI and ligated into the pGBKT7 bait vector (Takara Bio Europe/Clontech, Saint-Germain-en-Laye, France). The yeast bait strain Y2HGold was transformed with the bait construct and the expression of Mtk was confirmed by sodium dodecylsulfate polyacrylamide gel electrophoresis (SDS-PAGE) and western blotting. SDO/–Trp/X/A plates were used for the auto-activation test. SDO/–Trp plates were used to compare the growth of yeast cells transformed with pGBKT7::Mtk or the pGBKT7 empty vector.

**Table 1:** Primer sequences used in this study.

Gene	Sequence (5′ > 3′)	
Mtk* (F2H)	Fwd	CTCAAGCTTCGAATTCCATCGTCACCAGGGACCC
	Rev	TAGATCCGGTGGATCCTTAATAAATTGGACCCGGTC
SdhB (F2H)	Fwd	CTCAAGCTTCGAATTCATGCGGCCGGGGGTTCAG
	Rev	TAGATCCGGTGGATCCTTATAACTTGGAACCGCTGT
Mtk* (BiFC)	Fwd	CATGGAGGCCGAATTCCATCGTCACCAGGGACCCATTTTC
	Rev	GCAGGTCGACGGATCCTTAATAAATTGGACCCGGTCTTGG
SdhB-YC & SdhB-YN	Fwd	CGCCACTAGTGGATCCATGAACACCCTTGCCTGC
	Rev	CATCCCGGGAGCGGTACCGTTACCGAAAGCCATCTGC
YC-SdhB &YN-SdhB	Fwd	CGCCACTAGTGGATCCAACACCCTTGCCTGCTTG
	Rev	ATTGAGCTGGGAGCGGTACCGTTACCGAAAGCCATCTGC

^*^We used the HRH isoform of Mtk in this study.

### Construction of the *F. graminearum* cDNA library

The Make Your Own Mate & Plate™ library system (Takara Bio Europe/Clontech) was used to construct the *F. graminearum* cDNA library. Briefly, total RNA was extracted from a fresh culture of *F. graminearum* using the RNeasy Kit (Qiagen, Manchester, UK). First-strand cDNA was amplified from total RNA using the SMART™ cDNA Synthesis Kit (Takara Bio Europe/Clontech). A long-distance PCR with adapter primers (Takara Bio Europe/Clontech) was used to synthesize double-stranded cDNA, which was then normalized using the Evrogen Trimmer-direct cDNA Normalization Kit (BioCat, Heidelberg, Germany) and purified using CHROMA SPIN TE-400 columns (Takara Bio Europe/Clontech). Purified double-stranded cDNA was linked to the GAL4 AD and a hemagglutinin (HA) epitope tag in the vector pGADT7-Rec (Takara Bio Europe/Clontech) by homologous recombination in the *S. cerevisiae* prey strain Y187 (Takara Bio Europe/Clontech). Transformed cells were selected on SDO/–Leu plates and the cell density was adjusted to >2 × 10^7^ cells/mL.

### Yeast two-hybrid assay

Yeast two-hybrid assays were carried out using the Matchmaker™ Gold Yeast Two-Hybrid System (Takara Bio Europe/Clontech). Co-transformed yeast cells containing the vectors pGBKT7–53 (encoding the GAL4 DNA-BD fused to murine p53) and pGADT7-T (encoding the GAL4 AD fused to the SV40 T-antigen) were used as positive controls, whereas those containing vector pGBKT7-Lam (encoding the GAL4 DNA-BD fused with human lamin C) and pGADT7-T were used as negative controls. The yeast strain Y2HGold was transformed with the pGBKT7::Mtk expression construct using a modified lithium acetate method (Takara Bio Europe/Clontech) and the cells were spread onto SDO/–Trp plates. Y2H/BD-Mtk was subsequently mated in 2× YPDA (Takara Bio Europe/Clontech) with the pre-transformed library. After 3–5 days, the putative interacting clones were selected on DDO/X/A. The selected clones were re-plated on QDO/X/A. The blue colonies were selected as positive interactions and the vectors were rescued in *Escherichia coli* XL1-blue competent cells. The inserts were verified by sequencing. The corresponding bait and prey vectors were mated again and plated onto high-stringency QDO/X/A medium to identify genuine interactions. The Y2HGold cells were then co-transformed with pGADT7::candidate prey and the pGBKT7 empty vector to verify that no colonies grew on the QDO/X/A plates. We also tested the candidate prey for auto-activation (see above).

### F2H assay

F2H assays were carried out using the F2H^®^-Kit Basic (Chromotek, Planegg-Martinsried, Germany). The Mtk coding region was amplified from *D. melanogaster* DNA using primers designed with the Clontech Primer Design tool (Table 1). The PCR product was digested with EcoRI and BamHI and ligated into vector Evrogen pTagGFP_2_ (BioCat, Barcelona, Spain). The SdhB coding region was amplified from *F. graminearum* cDNA using the primers shown in Table 1. The PCR product was digested with the same enzymes as above and ligated into vector Evrogen pTagRFP (BioCat). Genetically modified BHK-21 cells (F2H^®^ cells, BioCat) were co-transfected with the pTagGFP_2_ and pTagRFP constructs. After 24 h, the co-transfected cells were fixed in 4% paraformaldehyde (Sigma-Aldrich, St Louis, MO, USA) and mounted on slides using Roti^®^-Mount FluorCare 4’,6-diamidino-2-phenylindole (DAPI) mounting medium (Carl Roth, Karlsruhe, Germany). Fixed cells were visualized using a Leica DMI 6000 B fluorescence microscope (Leica Microsystems, Wetzlar, Germany).

### BiFC assay

The Mtk coding region was amplified from *D. melanogaster* DNA as above. The PCR product was digested with BamHI and KpnI and ligated into the pNBV vectors in all possible orientations, as previously described^17, 18^. The in-frame cloning of the bait protein (Mtk) and fluorescent protein (YFP) was confirmed by sequencing. The SdhB coding region was amplified from *F. graminearum* cDNA using primers designed with the Primer Design tool (Table 1). Bait and prey constructs in vector pNBV were isolated by digestion with NotI and transferred to vector pMLBART. The resulting pMLBART plasmids were introduced into *A. tumefaciens* strain GV3101 and injected into the leaves of *Nicotiana benthamiana* plants (3–4 weeks old) as previously described^17^. Three days after injection, discs from the infected leaf tissue were excised and stained with 100 ng/mL DAPI (Sigma-Aldrich). The discs were examined using a Leica DM5500 B microscope and LAS v4.3 software (Leica Microsystems) with an exposure time of 512 ms for the detection of YFP fluorescence.

### Mitochondrial complex II activity

The mitochondrial complex II (SDH) activity of *F. graminearum* and *P. indica* was measured using the Succinate Dehydrogenase Activity Colorimetric Assay Kit (BioVision, Milpitas, CA, USA). Briefly, fungal mycelia were placed in 250-mL flasks containing 100 mL of potato dextrose broth at 24 °C, shaking at 130 rpm, in an incubator for 48 h. The mycelia were collected and washed twice with 0.9% NaCl, passed through Miracloth (EMD Millipore, Massachusetts, USA), and 0.2-g samples of fresh mycelia were incubated with Trichoderma lysing enzyme (Sigma-Aldrich) at 37 °C for 2 h with gentle shaking. The solution was then filtered to remove the mycelia. Enzymatic activity was stopped by washing the protoplasts twice in STC buffer (10 mM Tris-HCl, 0.8 M sorbitol, 80 mM CaCl_2_, pH 7.5) and resuspending them in cold isolation buffer (10 mM Tris-HCl, 0.4 M mannitol, 1 M EDTA, 0.4% bovine serum albumin (BSA), 0.3 mM phenylmethylsulfonyl fluoride, pH 7.2). Protoplasts were homogenized in a Dounce homogenizer (20× strokes), diluted 1:1 with washing buffer (10 mM Tris-HCl, 0.6 M mannitol, 1 mM EDTA, 0.4% BSA, pH 7.2) and centrifuged for 13 min at 1600 g. The supernatant was collected and centrifuged for 20 min at 7000 g. The mitochondrial pellet was washed twice with washing buffer, and SDH activity was detected after adding different concentrations (0, 0.06, 0.12, 0.25, 0.5, 1 and 2 µM) of synthetic Mtk (Pepmic Suzhou, Jiangsu, China) in a 96-well plate (Greiner Bio One, Frickenhausen, Germany) according to the instructions in the assay kit. We prepared a standard curve by adding 0, 4, 8, 12, 16 and 20 μl of the 2 mM DCIP standard into a series of wells in 96-well plate to yield standards of 0, 8, 16, 24, 32 and 40 nmol/well. We then added 50 μl of the reaction mix (46 μl SDH Assay Buffer, 2 μl SDH Substrate Mix, 2 μl SDH Probe) to each well. The absorbance at 600 nm was measured immediately in kinetic mode for 30 min at 25 °C. We then chose the start and end points in the linear range to calculate the SDH activity of the samples (nmol/min/µl). To demonstrate inhibition, we presented the mitochondrial SDH activity of Mtk-treated samples relative to the activity of the control (0 µM peptide), which was set to 100%. The experiment was carried out three times.

### Sequence alignment and phylogenetic analysis

SdhB amino acid sequences were aligned using the conserved domain platform at http://www.ncbi.nlm.nih.gov/Structure/cdd/cdd.shtml^39, 40^ and analyzed using PSI-BLAST^41^. A comprehensive phylogenetic tree was constructed using iTOL v3.4.3^42^ on the EMBL-EBI platform (http://itol.embl.de/) with the SdhB sequences of selected Ascomycota and Basidiomycota (see the list of sequences in the Supplementary Information).

## Electronic supplementary material

Supplementary Information
