# Supplementary material for: The selective antifungal activity of Drosophila melanogaster metchnikowin reflects the species-dependent inhibition of succinate–coenzyme Q reductase
Source: Sci Rep. 2017 Aug 15;7:8192. doi: 10.1038/s41598-017-08407-x (PMC5557811; doi:10.1038/s41598-017-08407-x)
Supplement: Supplementary file 1 — Supplementary Information [file 41598_2017_8407_MOESM1_ESM.pdf]

The selective antifungal activity of *Drosophila melanogaster* metchnikowin reflects the species-dependent inhibition of succinate–coenzyme Q reductase

Mohammad-Reza Bolouri Moghaddam[1,2], Thomas Gross[3], Annette Becker[3], Andreas Vilcinskas[1,2,*], Mohammad Rahnamaeian[2,*]

[1] Institute for Insect Biotechnology, Justus Liebig University of Giessen, Heinrich-Buff-Ring 26-32, D-35392 Giessen, Germany

[2] Fraunhofer Institute for Molecular Biology and Applied Ecology, Department of Bioresources, Winchester Strasse 2, D-35394 Giessen, Germany

[3] Institute of Botany, Justus Liebig University of Giessen, Heinrich-Buff-Ring 38, D-35392 Giessen, Germany

[*] Shared senior authorship; correspondence may be addressed to mohammad.rahnamaeian@uni-giessen.de or andreas.vilcinskas@agrar.uni-giessen.de

Email addresses:

Mohammad Reza Bolouri Moghaddam: mohammad.r.bolouri-moghaddam@bio.uni-giessen.de

Thomas Gross: thomas.gross@bio.uni-giessen.de

Annette Becker: annette.becker@bot1.bio.uni-giessen.de

Andreas Vilcinskas: andreas.vilcinskas@agrar.uni-giessen.de

Mohammad Rahnamaeian: mohammad.rahnamaeian@uni-giessen.de

Fig. S1. Testing the interaction between Mtk and SdhB in tobacco cells.

The SdhB fusion constructs showed no YFP fluorescence in the presence of YFP_{Cempty} (YCe) or YFP_{Nempty} (YNe). However, the Mtk constructs exhibited YFP fluorescence in both the cytoplasm and nucleus in the presence of the corresponding YFP construct without a fusion. The co-expression of Mtk and SdhB did not result in detectable YFP fluorescence, indicating that the BiFC assay was not suitable for the verification of interactions between Mtk and SdhB. (a) DAPI channel; (b) GFP channel.

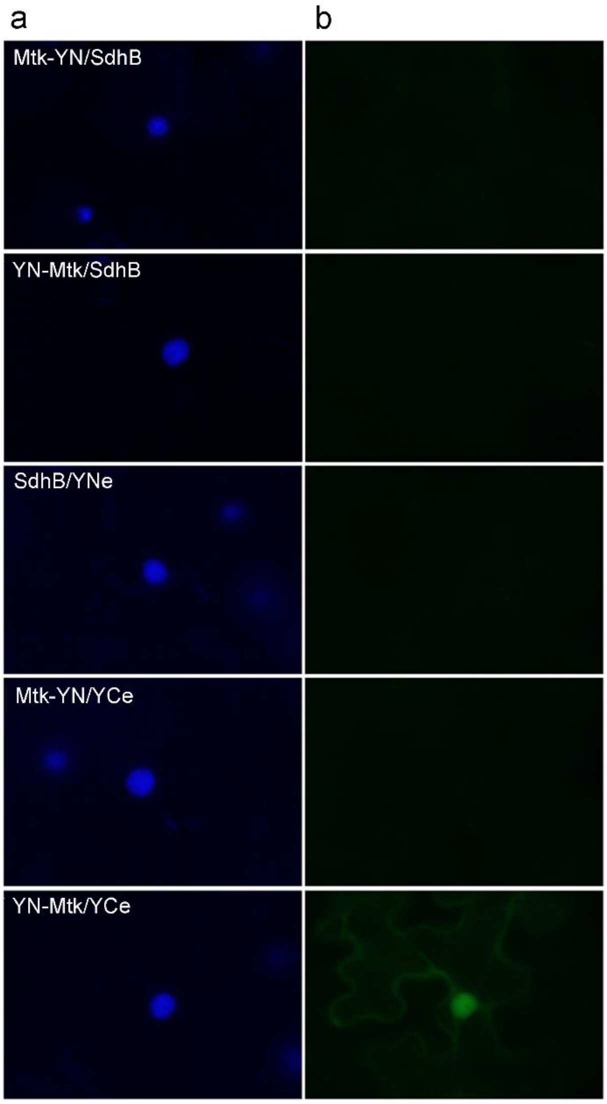

List of UniProtKB accession numbers of SdhB sequences in fungi for phylogenetic analysis.

Ascomycota:

Q2TWM0 Aspergillus oryzae; Q5BAU8 Emericella nidulans; Q0UY13 Phaeosphaeria nodor; A3M027 Scheffersomyces stipites; I1RNM7 Gibberella zeae (Fusarium graminearum); G8ZYS0 Torulaspora delbrueckii; A0A0F8BTS8 Ceratocystis fimbriata f. sp. platani; A0A0E9NA54 Saitoella complicate; J3P9K0 Gaeumannomyces graminis var. tritici; A5E0Y7 Lodderomyces elongisporus; F7VKJ3 Sordaria macrospora; A1DEE7 Neosartorya fischeri; C4JPG2 Uncinocarpus reesii; G2Q420 Myceliophthora thermophile; G8JXV6 Eremothecium cymbalariae; A5DJ38 Meyerozyma guilliermondii; B2AXV1 Podospora anserine; D5G7W1 Tuber melanosporum; G2RFL3 Thielavia terrestris; C4Y771 Clavispora lusitaniae; K3VJU5 Fusarium pseudograminearum; G2X2B4 Verticillium dahlia; K1WHZ3 Marssonina brunnea f. sp. multigermtubi; R8BC23 Togninia minima; M7T7N1 Eutypa lata; G0SBU7 Chaetomium thermophilum; M2MY09 Baudoinia compniacensis; R1GBW3 Botryosphaeria parva; G1X793 Arthrobotrys oligospora; C5FS39 Arthroderma otae; E4ZZI8 Leptosphaeria maculans; M2SI37 Cochliobolus sativus; F0XF17 Grosmannia clavigera; G0V5W8 Naumovozyma castellii; M3D0M2 Sphaerulina musiva; E3Q6P3 Colletotrichum graminicola; S8C423 Dactylellina haptotyla; W6ZHQ3 Bipolaris oryzae; R7YXR2 Coniosporium apollinis; G0WAJ7 Naumovozyma dairenensis; K2SD71 Macrophomina phaseolina; R0I5M2 Setosphaeria turcica; A0A084GGK4 Scedosporium apiospermum; W3XRT1 Pestalotiopsis fici; A0A0V1Q513 Debaryomyces fabryi; M1W191 Claviceps purpurea; A0A0D2IAT0 Rhinocladiella mackenziei; W9XQ08 Capronia epimyces; A0A0D2A4H2 Verruconis gallopava; W2RQM1 Cyphellophora europaea; I2H497 Tetrapisispora blattae; H2B1Y0 Kazachstania Africana; R9XB73 Ashbya aceri; A0A0X8HT74 Eremothecium sinecaudum; U4KU36 Pyronema omphalodes; V5FBB5 Byssochlamys spectabilis; M2WK03 Dothistroma

septosporum; S3C661 Ophiostoma piceae; A0A084QTY5 Stachybotrys chlorohalonata; A0A0G2DXW8 Phaeomoniella chlamydospora; 0A0D1WTD3 Exophiala Sideris; A0A0F7ZS57 Hirsutella minnesotensis; A0A0A1T5X1 Torrubiella hemipterigena; A0A0P7BGU0 Neonectria ditissima; A0A086T0I5 Acremonium chrysogenum; W9C5Z8 Sclerotinia borealis; A0A0C3H6Q6 Oidiodendron maius; A0A0F9X6K7 Trichoderma harzianum; A0A063C926 Ustilaginoidea virens; A0A136JFM4 Microdochium bolleyi; A0A0G2I336 Emmonsia crescens; A0A139HZQ5 Pseudocercospora musae; W6MMZ3 Kuraishia capsulate; N1J5Q8 Blumeria graminis f. sp. hordei; A0A0F4ZJL3 Thielaviopsis punctulata; Q6MVG9 Neurospora crassa; A0A0L1HS04 Stemphylium lycopersici; A0A0B1PFX7 Uncinula necator; G8EI90 Zymoseptoria tritici; A0A1E3Q0M3 Lipomyces starkeyi; A0A168CFB2 Isaria fumosorosea; A0A0D9MRU9 Aspergillus flavus; F8N0V7 Neurospora tetrasperma; A0A0A2VID1 Beauveria bassiana; A0A1J9QJH1 Emergomyces pasteuriana; A0A139GWE5 Mycosphaerella eumusae; A0A0A8L5G6 Kluyveromyces dobzhanskii; A0A060T220 Blastobotrys adeninivorans; A0A0U5B1X1 Passalora nattrassii; A0A165K4D7 Xylona heveae; W9JVT9 Fusarium oxysporum; A0A194VQF5 Valsa mali; A0A0G4KTR3 Verticillium longisporum; A7TNK6 Vanderwaltozyma polyspora; A0A1E3PTT9 Nadsonia fulvescens var. elongate; S3CSY3 Glarea lozoyensis; A0A1E3NR25 Pichia membranifaciens; A0A1E3QSX5 Babjeviella inositovora; A0A125RKY8 Pyrenophora teres f. teres A0A093VHK0 Talaromyces marneffei; A0A1L7WEU8 Phialocephala subalpine; A0A179FS63 Pochonia chlamydosporia; A0A0J9XIV1 Geotrichum candidum; R4XFE7 Taphrina deformans; L0PD02 Pneumocystis jiroveci; A0A1L9SUM9 Penicilliopsis zonata; A0A0J0ADI9 Gibberella fujikuroi; A0A177ADU6 Pseudogymnoascus destructans; A0A1E4TJV0 Tortispora caseinolytica; A0A1E4RWU9 Cyberlindnera jadinii; T5BQ28 Blastomyces dermatitidis; A0A1E3P4I6 Wickerhamomyces anomalus; S4SLP3 Monilinia fructicola; B6F0Y3 Aspergillus oryzae; A0A1G4JXZ6 Lachancea mirantina; A0A178BAK5 Stagonospora sp.; A0A151GIL0 Drechmeria coniospora; A0A117E0M6 Aspergillus niger;

N1NZC0 Saccharomyces cerevisiae; A0A0L0NE48 Tolypocladium ophioglossoides; A0A177BZZ3 Paraphaeosphaeria sporulosa; A0A1C1XRU7 Diaporthe helianthi; A0A061HP54 Blumeria graminis f. sp. tritici; A0A167XWF0 Aschersonia aleyrodis; A0A0M9VT85 Escovopsis weberi; A0A1A0HEA7 Metschnikowia bicuspidata var. bicuspidate; A0A1B8AK78 Fusarium poae; A0A022WBT3 Trichophyton rubrum; A0A167H468 Cordyceps brongniartii; A0A150V0F8 Acidomyces richmondensis; A0A1J9QWG5 Diplodia corticola; A0A0C4DT89 Magnaporthiopsis poae; A0A175VZ01 Madurella mycetomatis; A0A0H4UQS7 Venturia inaequalis; A0A1A7MP27 Aureobasidium pullulans; G4W729 Stagonosporopsis cucurbitacearum; A0A0B4UGK3 Alternaria alternate; E0D565 Podosphaera xanthii; A0A179I0W7 Purpureocillium lilacinum; A0A1D8NFY4 Yarrowia lipolytica; G8AE23 Millerozyma farinose; A0A0B7JM52 Bionectria ochroleuca; A0A089WYW9 Botryotinia fuckeliana; E0D564 Podosphaera xanthii; A0A167F5K0 Sugiyamaella lignohabitans; A0A0B4FV24 Metarhizium anisopliae; A0A1E4SKL1 Candida tanzawaensis; N4U420 Fusarium oxysporum f. sp. cubense; A0A178EBJ2 Pyrenochaeta sp.; A0A0N0NLZ9 Phialophora attae; A0A179UJL1 Ajellomyces dermatitidis; A0A0F2M4G5 Sporothrix schenckii; A0A0K2QQ30 Trichophyton mentagrophytes; A0A1L0AWJ4 Hanseniaspora guilliermondii; A0A167V949 Ascosphaera apis; A0A1D2VLH6 Ascoidea rubescens; A0A0J5PVK1 Aspergillus fumigatus; A0A1P8VHT0 Magnaporthe oryzae; C0SFU8 Paracoccidioides brasiliensis; A0A024S1K3 Hypocrea jecorina; A0A178Z551 Fonsecaea erecta; A0A1B7SIZ0 Ogataea polymorpha; A0A1J7J773 Coniochaeta ligniaria; A0A1E1MVM0 Rhynchosporium secalis; A0A1D5AKD3 Didymella tanaceti; A0A166LCX0 Colletotrichum incanum; A0A1Q5UJH8 Penicillium subrubescens; S5U054 Alternaria solani; A0A1B2JGC3 Komagataella pastoris; A0A084RG07 Stachybotrys chartarum; A0A1E5RDC2 Hanseniaspora uvarum; D5MTG0 Corynespora cassiicola; A0A1E4TQ48 Pachysolen tannophilus; A0A0F4YFV6 Rasamsonia emersonii.

Basidiomycota:

P32420 Ustilago maydis; Q70KF8 Uromyces fabae; G7E3H2 Mixia osmundae; A0A067N939 Pleurotus ostreatus; A0A0F7S7G2 Sporisorium scitamineum; D8PXF2 Schizophyllum commune; F4RTE2 Melampsora larici-populina; K5WSH2 Phanerochaete carnosa; E2RZU1 Helicobasidium mompa; R9AD16 Wallemia ichthyophaga; S7PV57 Gloeophyllum trabeum; W4JQS4 Heterobasidion irregular; A0A061HAV5 Anthracocystis flocculosa; S8DUS9 Fomitopsis pinicola; V5F1L5 Kalmanozyma brasiliensis; A0A0P9EXB3 Rhodotorula graminis; F8P9Z8 Serpula lacrymans var. lacrymans; A0A0C3SF13 Phlebiopsis gigantean; M2QHB0 Ceriporiopsis subvermispora; A0A0J0XIQ7 Cutaneotrichosporon oleaginosus; A0A0H2RKC8 Schizopora paradoxa; A0A067PGQ6 Jaapia argillacea; A0A0C2W5R2 Amanita muscaria Koide; A0A067SH62 Galerina marginata; A0A067M073 Botryobasidium botryosum; A0A0C9VSH8 Hydnomerulius pinastri; A0A0C3B1W4 Piloderma croceum; A0A0D0AUY5 Suillus luteus; A0A0C9XK69 Laccaria amethystine; M5FR71 Dacryopinax primogenitus; A0A0D7BRQ6 Cylindrobasidium torrendii; A0A0L6WZZ0 Termitomyces sp.; A0A0C3Q0Z8 Tulasnella calospora; A0A0C3B6Q8 Serendipita vermifera (Piriformospora indica); A0A0D7A300 Fistulina hepatica; A0A0F7SFL9 Phaffia rhodozyma; A0A0P1BA25 Ceraceosorus bombacis; O42714 Agaricus bisporus; A0A151VRY3 Hypsizygus marmoreus; A0A0B7FBK7 Thanatephorus cucumeris; Q76KC3 Lentinula edodes; C0MN02 Hebeloma cylindrosporum; A0A1I9UVP2 Agrocybe aegerita; U5H8G0 Microbotryum lychnidis-dioicae; A8Q7S4 Malassezia globose; J6F2W0 Trichosporon asahii var. asahii; A0A1J8PYA6 Rhizopogon vesiculosus; B1AC68 Coprinopsis cinerea; A0A173DSZ5 Ceratobasidium cereal; A0A0W0FH87 Moniliophthora roreri; A0A0C3E185 Scleroderma citrinum; A0A1E3K5W9 Tsuchiyaea wingfieldii; A0A0C9TL19 Paxillus involutus; A0A0D2NNG1 Hypholoma sublateritium; X8JUQ9 Rhizoctonia solani; M9MBS9 Pseudozyma Antarctica; A0A0C3NAI7 Pisolithus tinctorius; A0A165SY25 Neolentinus lepideus; A0A166E5N6 Peniophora sp.; A0A165CXP7 Laetiporus sulphureus; A0A137Q2D9 Leucoagaricus sp.; A0A165DMJ9 Exidia

glandulosa; A0A165REN6 Daedalea quercina; A0A165ZW91 Fibulorhizoctonia sp.; A0A180H0E4 Puccinia triticina; A0A166DA95 Sistotremastrum suecicum; A0A177V4H2 Tilletia walker; A0A165EHJ8 Calocera cornea; A0A077R4C7 Melanopsichium pennsylvanicum; G3LGR3 Ganoderma lucidum; C0STD8 Pholiota nameko; A0A061B326 Rhodosporidium toruloides; A0A146H2T9 Mycena chlorophos; A0A1B9GRB8 Kwoniella heveanensis; A0A1E3IUN1 Cryptococcus depauperatus; A0A1M2VTI8 Trametes pubescens; A0A1C7MPB6 Grifola frondosa; X8JV06 Rhizoctonia solani.
